# Safety and antitumor activity of GD2-Specific 4SCAR-T cells in patients with glioblastoma

**DOI:** 10.1186/s12943-022-01711-9

**Published:** 2023-01-09

**Authors:** Zhuohao Liu, Jiayi Zhou, Xinzhi Yang, Yuchen Liu, Chang Zou, Wen Lv, Cheng Chen, Kenneth King-yip Cheng, Tao Chen, Lung-Ji Chang, Dinglan Wu, Jie Mao

**Affiliations:** 1 Department of Neurosurgery, Shenzhen Hospital, Southern Medical University, Shenzhen, Guangdong China; 2 Shenzhen Key Laboratory of Viral Oncology, The Clinical Innovation & Research Centre, Shenzhen Hospital, Southern Medical University, Shenzhen, Guangdong China; 3 The Third School of Clinical Medicine, Southern Medical University, Shenzhen, Guangdong China; 4 Present Address: Department of Neurosurgery, Shenzhen People’s Hospital (The Second Clinical Medical College, Jinan University; The First Affiliated Hospital, Southern University of Science and Technology), Shenzhen, Guangdong China; 5 Shenzhen Geno-Immune Medical Institute, Shenzhen, Guangdong China; 6 School of Medicine, Life and Health Sciences, The Chinese University of Hong Kong (Shenzhen), Shenzhen, Guangdong China; 7 Department of Health Technology and Informatics, The Hong Kong Polytechnic University, Hong Kong, China

**Keywords:** GD2, 4SCAR-T, GBM, Safety, Tumor microenvironment

## Abstract

**Background:**

This study aimed to validate whether infusion of GD2-specific fourth-generation safety-designed chimeric antigen receptor (4SCAR)-T cells is safe and whether CAR-T cells exert anti-glioblastoma (GBM) activity.

**Methods:**

A total of eight patients with GD2-positive GBM were enrolled and infused with autologous GD2-specific 4SCAR-T cells, either through intravenous administration alone or intravenous combined with intracavitary administration.

**Results:**

4SCAR-T cells expanded for 1–3 weeks and persisted at a low frequency in peripheral blood. Of the eight evaluable patients, four showed a partial response for 3 to 24 months, three had progressive disease for 6 to 23 months, and one had stable disease for 4 months after infusion. For the entire cohort, the median overall survival was 10 months from the infusion. GD2 antigen loss and infiltrated T cells were observed in the tumor resected after infusion.

**Conclusion:**

Both single and combined infusions of GD2-specific 4SCAR-T cells in targeting GBM were safe and well tolerated, with no severe adverse events. In addition, GD2-specific 4SCAR-T cells partially mediate antigen loss and activate immune responses in the tumor microenvironment. Validation of our findings in a larger prospective trial is warranted.

**Trial registration:**

ClinicalTrials.gov Identifier: NCT03170141. Registered 30 May 2017.

## Background

Outcomes for patients with glioblastoma (GBM), the most aggressive and lethal human brain tumor, remain poor despite combined treatment including surgical resection, radiotherapy, and chemotherapy [1, 2]. Chimeric antigen receptor (CAR)-T therapy, which combines specific recognition of tumor antigens by monoclonal antibodies and the tumor-killing function of T cells, provides a new strategy for cancer treatment [3]. CAR-T therapy has shown great promise for hematologic cancers including non-Hodgkin’s lymphoma, chronic lymphocytic leukemia, and acute lymphocytic leukemia, leading to US Food and Drug Administration approval of four CAR-T products: Kymriah, Yescarta, Tecartus, and Breyanzi [3–6]. Recently, in the longest follow-up study for CAR-T therapy so far, 60% of B-cell lymphoma patients who underwent CAR-T therapy remained in remission at 5 years [7]. However, the challenges for CAR-T therapy for solid tumors continue, with only a few patients having a good prognosis [3, 8–10]. Previous studies have uncovered four main obstacles, including heterogeneous antigen expression, impaired CAR-T cell fitness, limited CAR-T cell homing and penetration, and an immunosuppressive microenvironment. Antigen selection is critical to CAR-T cell function, while heterogeneous antigen expression results in limited targetable antigens for CAR-T cells [11]. Sufficient effective CAR-T cell traffic to the tumor site is crucial for CAR-T therapy; however, impaired CAR-T cell fitness post infusion and limited CAR-T cell homing and penetration to solid tumors largely impede the tumor-killing efficacy [12, 13]. Immunosuppressive cells accumulate in the tumor microenvironment impairing the tumor-killing efficacy of CAR-T cells. In addition, immunosuppressive cells generate an environment hostile to CAR-T cells by secreting a variety of inhibitory cytokines [14, 15].

Immunotherapy with genetically modified T cells expressing different CARs is currently under investigation for GBM. Clinical trials with CAR-T cells targeting interleukin(IL)-13Rα2, epidermal growth factor receptor variant III (EGFRvIII), and human epidermal growth factor receptor 2 (HER2) for GBM treatment have been completed [16–18]. Although the safety of CAR-T cells for GBM treatment has been proven in these three trials, CAR-T cells as a monotherapy are not particularly effective in GBM due to antigen escape and tumor heterogeneity. In addition to IL-13Rα2, EGFRvIII, and HER2, various targets have been identified for CAR-T therapy for GBM. GD2 disialoganglioside is primarily expressed on the cell membrane of neurons in normal tissue and also highly expressed in many malignant tumors. In brain tumors, GD2 is enriched on the tumor surface of 80% of diffuse intrinsic pontine glioma (DIPG) [19]. Functionally, GD2 exhibits cancer stem cell (CSC) properties in breast cancer and is specifically overexpressed in GBM CSCs, though it did not show potential as a therapeutic target for GBM CSCs [20, 21]. Animal studies have shown that CAR-T cells against GD2 can effectively eliminate GD2-positive human GBM and DIPG tumors implanted orthotopically in mice without obvious neurotoxicity or off-target effects [19, 22, 23]. Recently, a clinical experience from four patients with K27M mutation in genes encoding histone H3 (H3K27M)-mutated DIPG or spinal cord diffuse midline glioma (DMG) treated with GD2-specific CAR-T cells has been reported. These early results highlighted that GD2-specific CAR-T therapy for H3K27M-mutated DIPG and spinal DMG is safe and exhibits clinical benefits [24]. Considering that GD2 is expressed on normal neural tissues and anti-GD2 antibody treatment for neuroblastoma is associated with neuropathic pain, further clinical exploration of GD2-specific CAR-T cells for GBM is warranted.

To address the safety concerns and to improve the anti-tumor efficacy, we used a fourth-generation safety-designed CAR (4SCAR). The 4SCAR consisted of CD28 transmembrane and cytoplasmic domains, co-stimulatory 4-1BB intracellular TRAF binding domain, CD3z chain intracellular domain, and an inducible suicide caspase 9 gene [25–28]. In this study, we performed a phase 1 trial of infusing GD2-specific 4SCAR-T cells in patients with progressive GBM and we report on its safety, persistence, and tumor-killing efficacy.

## Methods

### Study design and participants

Patients were subjected to lymphodepletion with fludarabine (25 mg/m^2^) and cyclophosphamide (300 mg/m^2^) on days − 4, -3, and − 2 prior to 4SCAR-T cell infusion. Patients received intravenous administration of 4SCAR-T cells at 2.5 × 10^6^ per kg of body weight. The intravenous infusion dose was determined on the basis of our previous publications [25, 26, 28]. Patients received intracavitary administration of 4SCAR-T cells at 1.0 × 10^5^ per kg of body weight. The chosen dose for intracavitary infusion was within the range of cell doses administered i.c.v. in other brain tumor trials [16, 18, 24].

Histological assessment of GD2 expression in surgically resected GBM tissues (WHO IV glioma) was conducted using immunohistochemistry (IHC) as previously described [25]. IHC staining of GD2 in tumor specimen from patients was performed using an anti-GD2 antibody (catalog no. 554,272; BD Biosciences). The isotype control was stained with purified mouse IgG2a (catalog no. 400,202; BioLegend). Images were captured from stained slides using a Axio Vert.A1 microscope (Zeiss) and were analyzed by ZEN imaging software (Zeiss). The tumor grading and expression of GD2 antigen were assessed by two pathologists from the Department of Pathology at Shenzhen Hospital of Southern Medical University and Shenzhen Geno-immune Medical Institute who were blinded to the patient information. The GD2 intensity was compared with that from isotype controls and scored from 0 to 4 according to staining intensity and the percentage of positive cells. Patients with GD2-positive GBM were enrolled in this study. Written informed consent was obtained for CAR-T infusion. Upon enrollment, patients were subjected to magnetic resonance imaging (MRI) to assess their disease, and the manufacture of GD2-specific 4SCAR-T cells was initiated. Exclusion criteria included GD2-negative tumors; clinical decline; loss of interest; previous CAR therapy; severe cardiac dysfunction requiring intervention; uncontrolled infection; receipt of any anti-cytokine agents or corticosteroids; pregnancy or breastfeeding; primary immunodeficiency or autoimmune disease. Enrolled patients with evidence of progressive disease and indication for surgery underwent neurosurgery during which the Rickham device was implanted in the cavity of the tumor resection site, followed by intravenous infusion combined with intracavitary autologous GD2-specific 4SCAR-T cell infusion. Enrolled patients who did not undergo surgery received only intravenous infusion. The copy number of 4SCAR-T cells was determined after CAR-T cell infusion. IL-6, TNFα, and IFNγ levels were measured using enzyme-linked immunosorbent assay kits (R&D). Clinical response to 4SCAR-T cell infusion was evaluated by monitoring the adverse events and performing MRI before and after 4SCAR-T cell infusion. Disease response was defined as a partial response (PR; 30% decrease in the longest diameter of the tumor), progressive disease (PD; 20% increase in the measurement of tumor), or stable disease (SD; small changes that do not meet the criteria for PR or PD).

### Construction of 4SCAR-GD2 lentiviral vector

An NHP/TYF lentivector system was used for packaging 4SCAR-GD2 vector as previously described [3–5]. The DNA sequences of GD2 scFv were cloned into a pTYF lentiviral vector, followed by the lentiviral particle package. The GD2 CAR sequence was constructed with lentiviral 4SCAR, which incorporated the CD28 transmembrane and cytoplasmic domains, the co-stimulatory 4-1BB intracellular TRAF binding domain, the CD3z chain intracellular domain, and an inducible suicide caspase 9 gene.

### Production of GD2-specific 4SCAR-T cells

Autologous GD2-specific 4SCAR-T cells were manufactured from 2 to 5 × 10^9^ lymphocytes in the peripheral blood. To prepare clinical-grade GD2-specific 4SCAR-T cells, a standard operation protocol was established in compliance with good manufacturing and laboratory practice following the regulatory guidelines for cell and gene therapy products, as previously described [25, 29]. Peripheral blood buffy coats were collected from patients and peripheral blood mononuclear cells (PBMCs) were isolated using Ficoll-Paque plus (GE Healthcare). Lymphocytes were activated using phytohemagglutinin (5 µg/mL) for 2–3 days, after which the cells were maintained in TexMACS medium supplemented with human IL-2 (40 U/mL), IL-7 (20 U/mL), and IL-15 (10 U/mL). Activated T cells were transduced with GD2-specific 4SCAR vectors and expanded for six–ten days. After expansion, the cells were tested for sterility, transduction efficiency, and killing function.

### Assessment of cytotoxic effects of GD2-specific 4SCAR-T cells

Patient-derived primary GBM cells with high GD2 expression and nearly undetectable CD19 expression were transduced with a lentiviral wasabi green fluorescence reporter gene (target cells), plated into 48-well plate, and cultured at 37 °C in 5% CO_2_. Effector cells including GD2-specific CAR-T cells, control PBMCs, and non-specific 4SCAR-T cells (CD19) were added to the wells of target cells (effector: target ratio, 4:1). Target cell death was determined by fluorescence microscopy and photographed at 24, 48, and 72 h after co-culture.

### Measurement of CAR copy number

Peripheral blood samples were obtained prior to 4SCAR-T cell infusion and at the indicated time points post infusion (Day 7, 14, 21, and 28). Genomic DNA was extracted from peripheral blood using a genomic DNA purification kit (Promega). The copy number of 4SCAR-T cells was determined by real-time quantitative PCR (RT-qPCR), as previously described [29, 30].

### Multiplex immunofluorescence

Multiplex immunofluorescence staining of GD2, CD8, and CD163 in pre- and post-GD2 specific 4SCAR-T cell infusion specimens from Patient 01 was performed using a PANO 7-plex IHC kit (Panovue) as previously described [31]. After incubation with anti-GD2 (catalog no. 554,272; BD Biosciences), anti-CD8 (catalog no. 70,306; Cell signaling Technology), and anti-CD163 (catalog no. 93,498; Cell Signaling Technology) primary antibodies, horseradish peroxidase-conjugated secondary antibodies and a tyramide signal amplification fluorescence kit (Panovue) were applied. Nuclei were stained with DAPI, followed by scanning and multispectral images capture using the PanoVIEW vs200 slide scanner (Panovue), equipped with an Olympus 20×lens.

### Endpoints

The primary objective of this clinical trial was to validate the feasibility and safety of autologous GD2-specific 4SCAR-T cell infusion in patients with GBM. The secondary objective was to determine 4SCAR-T cell expansion, persistence, and anti-GBM effects.

### Statistics

Adverse events were recorded based on the number and proportion of patients who received GD2-specific CAR-T cell administration. Data are expressed as mean ± SEM, and statistical analysis was performed using SPSS or GraphPad Prism 8.0. The equality of variance was assessed using the Levene test.

## Results

### Clinical protocol design and patient characteristics

**Fig. 1:**
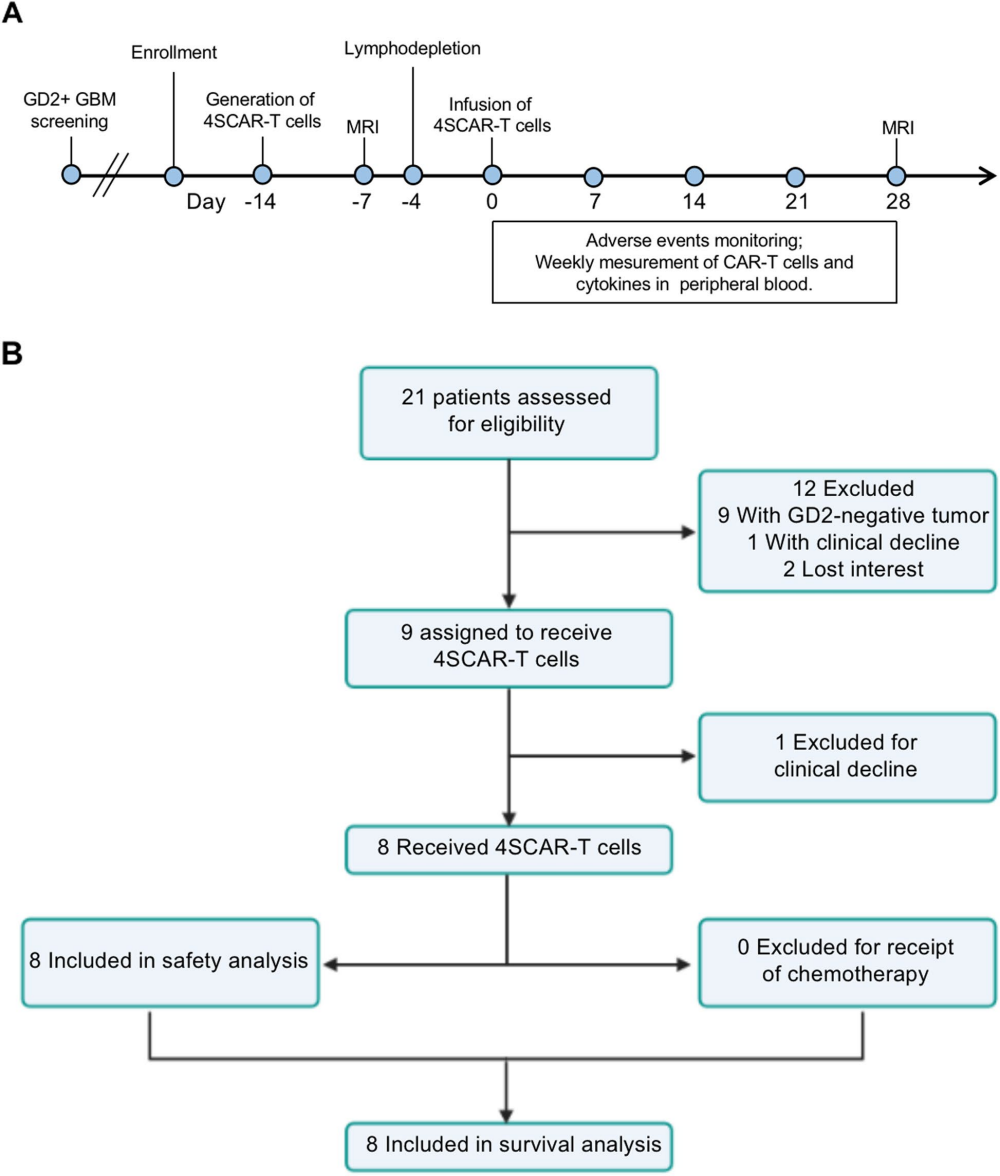
Protocol schema and consort flow diagram. **A** Protocol schema for GD2 testing, enrollment, generation of 4SCAR-T cells, lymphodepletion, 4SCAR-T cell infusion, and follow-up. **B** Consort diagram showing the number of patients assessed and enrolled on the study

We performed a phase 1 trial of infusing GD2-specific 4SCAR-T cells in patients with progressive GBM according to the clinical protocol design (Fig. 1A), to validate whether infusion of GD2-specific 4SCAR-T cells is safe and whether 4SCAR-T cells exert anti-GBM activity. IHC was performed to assess the GD2 expression in surgically resected GBM tissues from different patients. Patients with GD2-positive GBM were enrolled in this study. Written informed consent was obtained for 4SCAR-T infusion. Upon enrollment, patients were subjected to baseline MRI assessment, and the manufacture of GD2-specific 4SCAR-T cells was initiated. Patients were subjected to lymphodepletion on days − 4, -3, and − 2 prior to 4SCAR-T cell infusion. Patients received 4SCAR-T cell infusion on day 0. After GD2-specific 4SCAR-T cell infusion, the adverse events, CAR-T cell number and cytokine level in peripheral blood were monitored weekly. On day 28, patients were subjected to post-infusion MRI assessment.

A total of 21 patients (12 women and 9 men) with recurrent or progressive GBM were assessed for eligibility for the clinical trial between July 18, 2019, and December 1, 2021. Eight patients with GD2-positive GBM, as confirmed by IHC, were enrolled in this study (Fig. 1B and Table 1). Four of the eight patients were 18 years or older (median age, 35 years; range, 29–63 years). Four patients were younger than 18 years of age (median age, 5 years; range, 3–6 years). All enrolled patients underwent surgical resection, and three of eight patients underwent 2 surgical resections (Table 1). The median time from diagnosis to T-cell infusion was 7 months (range, 2–19 months). All enrolled patients had IDH1/2 wildtype. Unmethylated MGMT was observed in seven of the eight patients (Table 1).

**Table 1:** Patient characteristics

Patient No.	Age (y) /Sex	Diagnosis	Prior Treatment					CAR-T Cells Dose and Routes
			**Surgery**	**Radiation** **+ TMZ**	**IDH1/2**	**MGMT**	**GD2 Intensity**	
**01**	63/F	GBM	Yes,×2	TMZ, No Radiation	WT	UM	4.0+	i.v. 1.3 × 10^8^ i.c. 5 × 10^6^
**02**	4/M	GBM	Yes,×2	Radiation, No TMZ	WT	UM	3.0+	i.v. 6.4 × 10^7^ i.c. 2.6 × 10^6^
**03**	6/F	GBM	Yes	Yes	WT	UM	2.0+	i.v. 4.6 × 10^7^
**04**	38/F	GBM	Yes	Yes	WT	UM	2.5+	i.v. 1.4 × 10^8^
**05**	29/M	GBM	Yes,×2	Yes	WT	ML	2.0+	i.v. 1.6 × 10^8^ i.c. 6.4 × 10^6^
**06**	3/M	GBM	Yes	Radiation, No TMZ	WT	UM	3.5+	i.v. 3.8 × 10^7^
**07**	5/F	GBM	Yes	Radiation, No TMZ	WT	UM	1.5+	i.v. 3 × 10^7^
**08**	31/M	GBM	Yes	Yes	WT	UM	3.5+	i.v. 2.1 × 10^8^

*TMZ* Temozolomide, *WT* Wildtype, *UM* Unmethylated, *ML* Methylated, *i.v.* Intravenous, *i.c.* Intracavitary

### Characteristics of GD2-specific 4SCAR-T cells

**Fig. 2:**
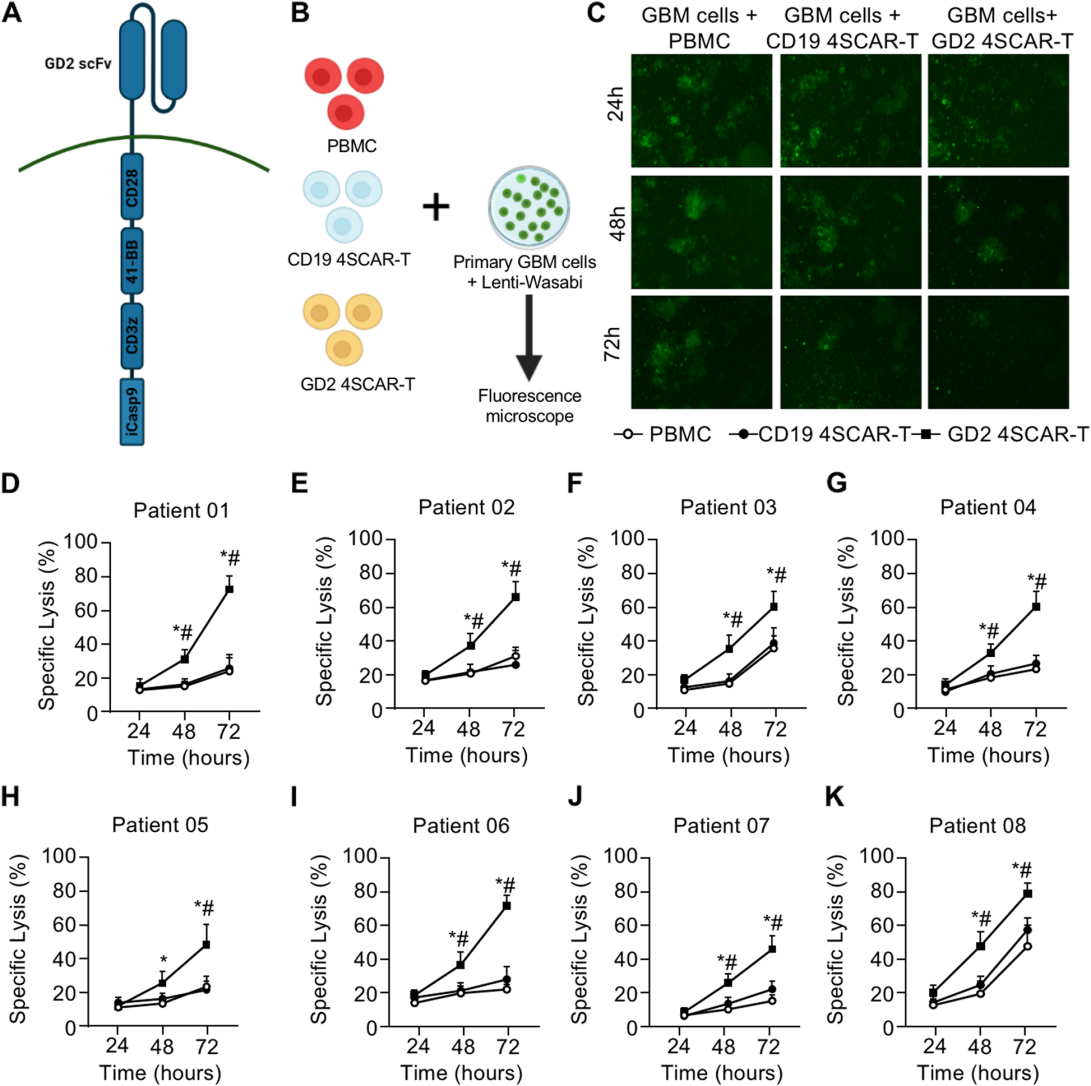
Design and killing assay of GD2 specific 4SCAR-T cells. **A** Design of GD2 specific 4SCAR-T cells. **B** Study design of killing assay of peripheral blood mononuclear cells (PBMCs), CD19 specific 4SCAR-T cells and GD2 specific 4SCAR-T cells against primary GBM cells. The primary GBM cells transduced with a lentiviral wasabi green fluorescence reporter gene were used as target cells, and plated into 48-well plate before co-culturing with different cells (E:T ratio 4:1). **C** Fluorescence microscopy of primary GBM cells from Patient 01 on 24, 48 and 72 h after co-culture. **D**-**K** The percentage of lysed primary GBM cells from indicated patients. All data are represented as the mean ± SEM. *p < 0.05, GD2 4SCAR-T vs. PBMC; #*p* < 0.05, GD2 4SCAR-T vs. CD19 4SCAR-T (One-Way ANOVA)

Autologous GD2-specific 4SCAR-T cells were manufactured from lymphocytes in the peripheral blood by transduction with GD2-specific 4SCAR vectors (Fig. 2A). The average transduction efficiency was 36.0% (range, 10.0-82.7%). Patient-derived primary GBM cells with lentiviral wasabi green fluorescent labeling were co-cultured with GD2-specific 4SCAR-T cells, control PBMCs, and non-specific 4SCAR-T cells (CD19) to assess their killing capacity (Fig. 2B). GD2-specific 4SCAR-T cells showed significant cytotoxic effects against primary GBM cells, when compared with PBMC and CD19-specific 4SCAR-T cells (Fig. 2C-K). Six of eight GD2-specific 4SCAR-T cells showed 60% or higher specific lytic activity of primary GBM cells at 72 h post co-culture (Fig. 2C-K).

### Safety and in vivo detection of GD2-specific 4SCAR-T cells

**Fig. 3:**
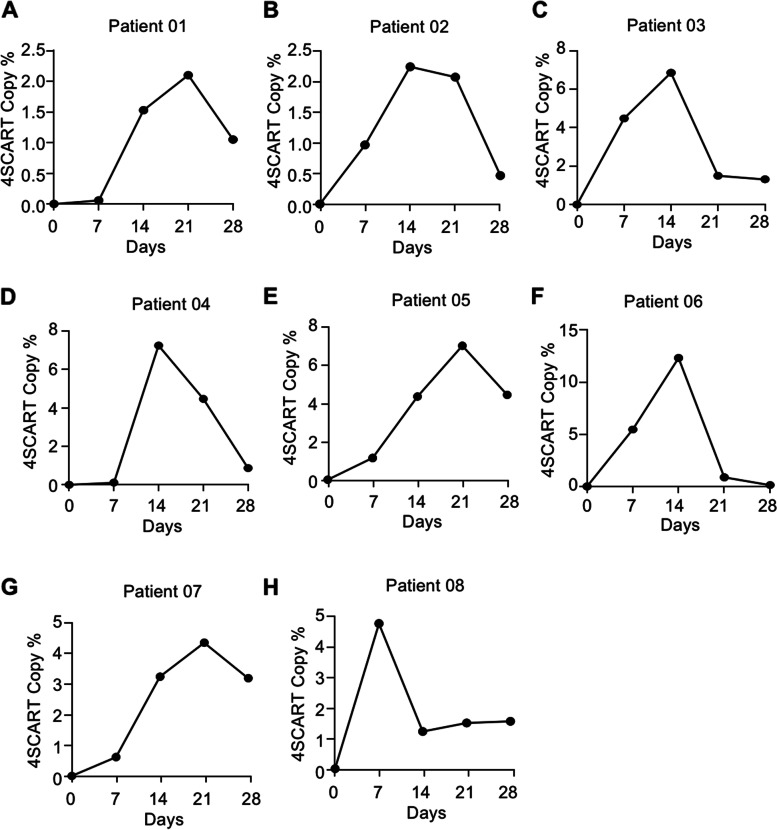
In vivo persistence of GD2-specific 4SCAR-T cells in peripheral blood. The copy number of GD2-specific 4SCAR-T cells in peripheral blood of indicated patients was determined by RT-qPCR analysis

To evaluate the safety of the delivery method of CAR-T cell infusion, either through the peripheral blood system or intracranial, the trial was designed with intravenous administration alone or intravenous combined with intracavitary administration. Eight patients received 11 infusions, with three patients receiving combined infusions (intravenous combined intracavitary administration; Table 1). Patient 01 had grade 2 seizures and grade 3 headache, which were probably related to T-cell infusion. No other CAR-T-related adverse events were observed (Table 2). The global score for health-related quality of life and scores on the other scales did not change significantly post GD2-specific 4SCAR-T cell infusion (Table 3). GD2-specific 4SCAR-T cell populations in peripheral blood were evaluated weekly by RT-qPCR in all patients after infusion. One patient (Patient 08) had the highest frequency of GD2-specific 4SCAR-T cells at 1 week after infusion (4.34%), four patients (Patient 02, 03, 04, and 06) had the highest frequency of GD2-specific 4SCAR-T cells at 2 weeks after the infusion (mean, 7.30%; range, 2.02–12.31%), and three patients (Patient 01, 05, and 07) had the highest frequency of GD2-specific 4SCAR-T cells at 3 weeks after the infusion (mean, 4.13%; range, 2.10–6.33%; Fig. 3A-H). At 4 weeks after infusion, GD2-specific 4SCAR-T cells were present in all patients (mean, 2.09%; range, 0.13–4.47%). These results indicate that GD2-specific 4SCAR-T cells can expand for 1–3 weeks and persist at a low frequency thereafter.

**Table 2:** Adverse events within the first 4 weeks after GD2-specific 4SCAR-T cell infusion

Adverse Event	No. of Patients (*n* = 8)			Adverse Event	No. of Patients (*n* = 8)		
	Garde 2	Grade 3	Grade 4		Garde 2	Grade 3	Grade 4
**Probably related**				**Respiratory**			
**Central nervous system**	0	0	0	**Atelectasis**	0	0	0
**Headache**	0	1	0	**Pain**			
**Seizure**	1	0	0	**Extremity**	0	0	0
**Unrelated**				**Bone**	0	0	0
**Hematologic toxic**				**Myalgia**	4	1	0
**Anemia**	2	0	0	**Musculoskeletal**			
**Lymphopenia**	4	1	0	**Edema, localized**	0	0	0
**Neutropenia**	5	0	0	**Fracture**	0	0	0
**Thrombocytopenia**	0	0	0	**Central nervous system**			
**Nonhematologic toxic**				**Headache**	1	1	0
**General**				**Seizure**	0	0	0
**Anorexia**	0	0	0	**Gait disturbance**	0	0	0
**Fatigue**	1	0	0	**Memory impairment**	0	0	0
**Somnolence**	1	0	0	**Tremors**	0	0	0
**Weakness**	1	0	0	**Cerebral edema**	0	0	0
**HEENT**				**Hydrocephalus**	0	0	0
**Eye paralysis, lateral**	0	0	0	**Infectious**			
**Gastrointestinal**				**Urinary tract infection**	0	0	0
**Nausea**	0	0	0	**Laboratory test results**			
**Diarrhea**	0	0	0	**Elevated ALT**	0	0	0
**Constipation**	0	0	0	**Elevated AST**	0	0	0
**Vomiting**	0	0	0	**Hyperbilirubinemia**	0	0	0
**Cardiac**				**Hyperkalemia**	0	0	0
**Bradycardia**	0	0	0	**Hypernatremia**	0	0	0
				**Hyponatremia**	0	0	0

**Table 3:** Scores for Health-Related Quality of Life post GD2 specific 4SCAR-T cell infusion

Measure	Score		*P* Value
	**Prior to infusion**	**Post infusion**	
**QLQ-C30**			
**Global**	51.0 ± 7.4	62.9 ± 14.8	0.062
**Physical functioning**	64.4 ± 7.6	65.0 ± 6.8	0.864
**Role functioning**	64.1 ± 6.1	63.4 ± 10.9	0.868
**Emotional functioning**	66.8 ± 13.8	67.8 ± 11.8	0.878
**Cognitive functioning**	56.1 ± 8.9	66.8 ± 11.1	0.053
**Social functioning**	62.6 ± 8.8	62.3 ± 5.4	0.920
**Fatigue**	35.6 ± 8.8	28.8 ± 10.3	0.174
**Nausea and vomiting**	4.1 ± 2.1	3.8 ± 1.8	0.709
**Insomnia**	18.1 ± 2.5	22.8 ± 5.8	0.058
**QLQ-b20**			
**Future uncertainty**	35.3 ± 5.6	29.6 ± 4.9	0.052
**Visual disorder**	18.3 ± 3.0	18.1 ± 2.5	0.929
**Motor dysfunction**	17.3 ± 2.4	19.3 ± 3.9	0.237
**Communication deficit**	21.5 ± 5.0	19.1 ± 3.6	0.295
**Bothered by hair loss**	5.8 ± 3.2	4.6 ± 2.9	0.468

### Tumor response and survival after infusion of GD2-specific 4SCAR-T cells

**Fig. 4:**
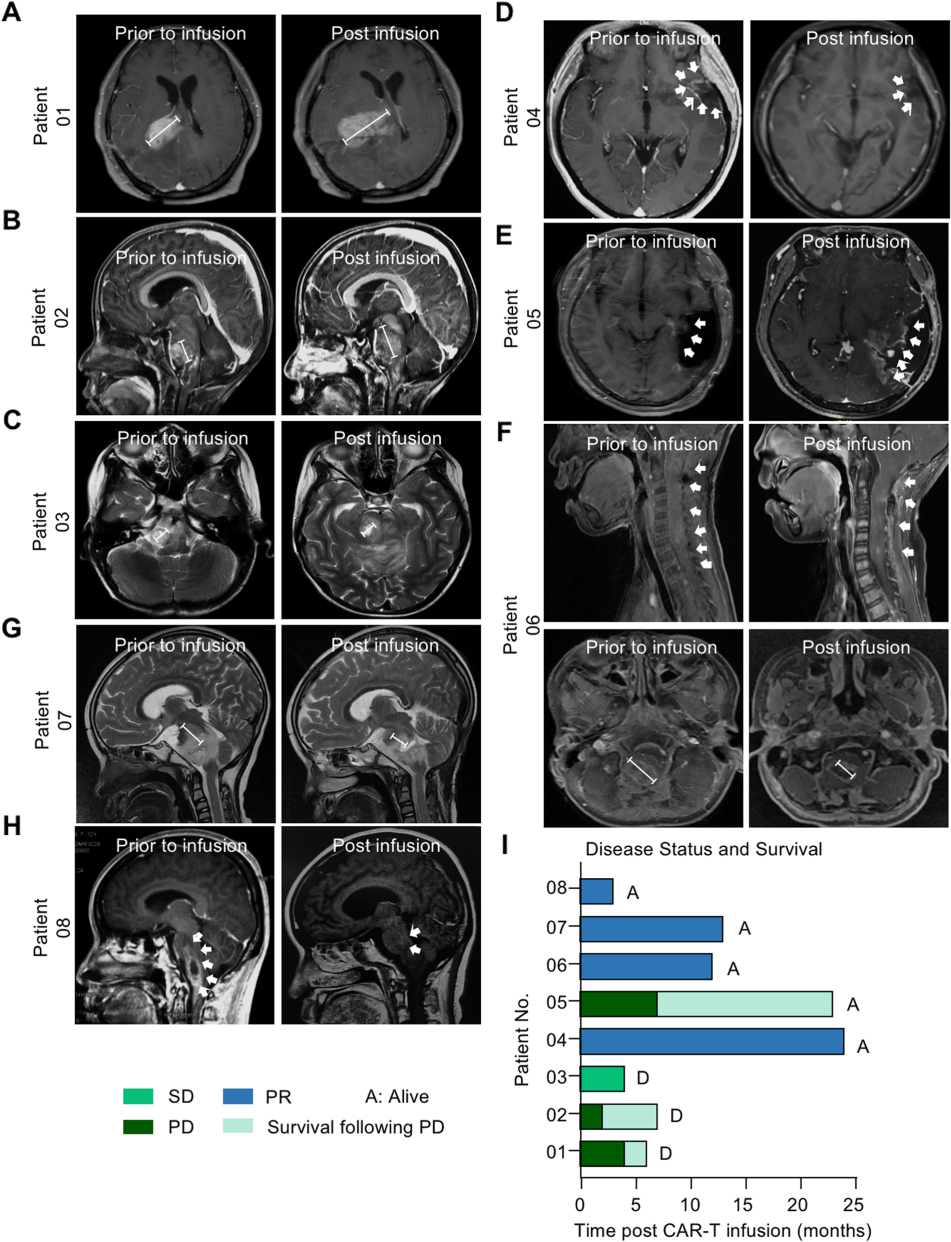
MRI scan of brain and overall survival post GD2 specific 4SCAR-T cell infusion. **A**-**H** Magnetic resonance imaging (MRI) scan of the brain before and 4 weeks after GD2 specific 4SCAR-T cell infusion. **I** Swimmer’s plot describing disease status and overall survival for each patient. PD, progressive disease. SD, stable disease. PR, partial response. A, Alive. D, Dead

To evaluate the GBM-killing activity of GD2-specific 4SCAR-T cells, MRI of the brain was performed 4 weeks after 4SCAR-T cell infusion (Fig. 4A-H). For the entire cohort, the median overall survival was 10 months from 4SCAR-T cell infusion (3–24 months, (Fig. 4I and Table 4). Of the eight evaluable patients, four (50%; Patient 04, 06, 07, and 08) had a partial response without further therapy after a single infusion of 4SCAR-T cells. MRI showed that the tumor size of these four patients was significantly decreased after 4SCAR-T infusion (Fig. 4D, F, G and H). Patient 04, a 38-year-old woman with GBM, received GD2-specific 4SCAR-T cells (i.v., 1.4 × 10^8^ cells) and had a partial response that lasted for 24 months (Fig. 4D and I). Of the eight evaluable patients, one patient (12.5%; Patient 03) had stable disease after 4SCAR-T cell infusion (Fig. 4C). However, Patient 03 died from hydrocephalus-induced brain herniation 4 months after 4SCAR-T cell infusion. Although three patients (37.5%; Patient 01, 02, and 05) had a progressive disease, all patients survived for more than 6 months (Fig. 4A, B, E, I, and Table 3). Although Patient 01 died of disease 6 months post 4SCAR-T infusion, Patient 02 and Patient 05 were still alive following disease progression without further therapy after a combined infusions of 4SCAR-T cells (Patient 02, 7 months; Patient 05, 23 months).

**Table 4:** Patient outcomes

Patient No.	Disease Response	Time to Progression (m; from infusion)	Survival (m)		Outcome
			**From diagnosis**	**From infusion**	
**01**	PD	4	12	6	DOD
**02**	PD	2	26	7	DOD
**03**	SD	No progression	11	4	Dead from brain herniation
**04**	PR	No progression	35	24	Alive
**05**	PD	7	29	23	Alive
**06**	PR	No progression	23	12	Alive
**07**	PR	No progression	15	13	Alive
**08**	PR	No progression	7	3	Alive

*M* Months, *SD* Stable disease, *PD* Progressive disease, *PR* Partial response, *DOD* Died of disease

### In situ immune modulation and cytokine modulation in peripheral blood

**Fig. 5:**
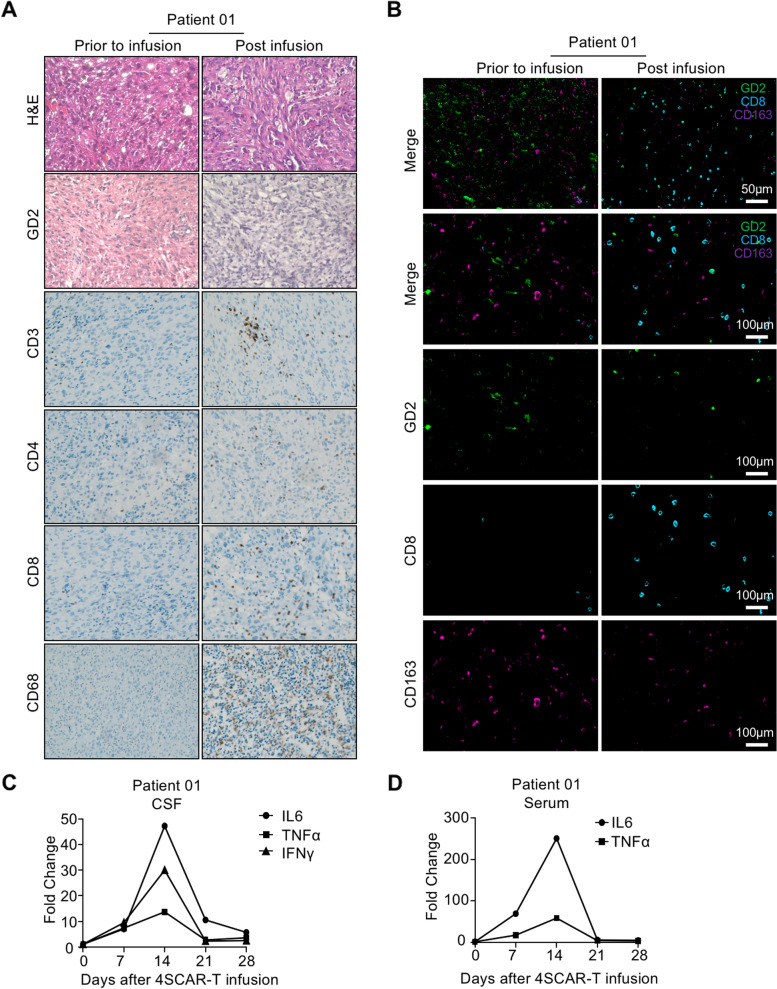
Immune modulation after GD2 specific 4SCAR-T cell infusion. **A** Hematoxylin and eosin (H&E) staining, IHC staining for GD2, CD3, CD4, CD8 an CD68 in pre- and post-GD2 specific 4SCAR-T cell infusion specimens from Patient 01. **B** Multiplex immunofluorescence staining of GD2, CD8 and CD163 in pre- and post-GD2 specific 4SCAR-T cell infusion specimens from Patient 01. **C** Circulating levels of IL-6, TNF-α, and IFNγ in cerebrospinal fluid (CSF) from Patient 01. (D) Circulating levels of IL-6 and TNF-α in serum from Patient 01

Patient 01, who had worsening contrast enhancement, underwent resection 6 weeks after infusion, providing us with an opportunity to explore the pathological status and immune microenvironment of the tumor. We detected the expression of GD2 antigen and infiltration of immune cells in the cancer lesions. Interestingly, we found that GD2 expression dramatically decreased after infusion (Fig. 5A and B). In addition, we detected a large number of T cells and macrophages in situ (Fig. 5A). More CD8 + T cells were found among infiltrated T cells than in pre-infusion tumor specimens from the same patient (Fig. 5A and B). Additionally, we confirmed that CD163 + M2 macrophages were less infiltrated in post-infusion tumor specimens, highlighting that GD2-specific 4SCAR-T cells remodel M2 macrophage-mediated suppressive immune microenvironment (Fig. 5B). Circulating levels of the inflammatory cytokines IL-6, TNFα, and IFNγ in the cerebrospinal fluid increased dramatically from pre-infusion baseline levels and peaked at 2 weeks after infusion (Fig. 5C). Consistently, increase in the levels of IL-6 and TNFα were observed in the serum (Fig. 5D). Hence, we concluded that the worsening contrast enhancement of Patient 01 reflected the immune-related effects of 4SCAR-T cells rather than true GBM progression.

**Fig. 6:**
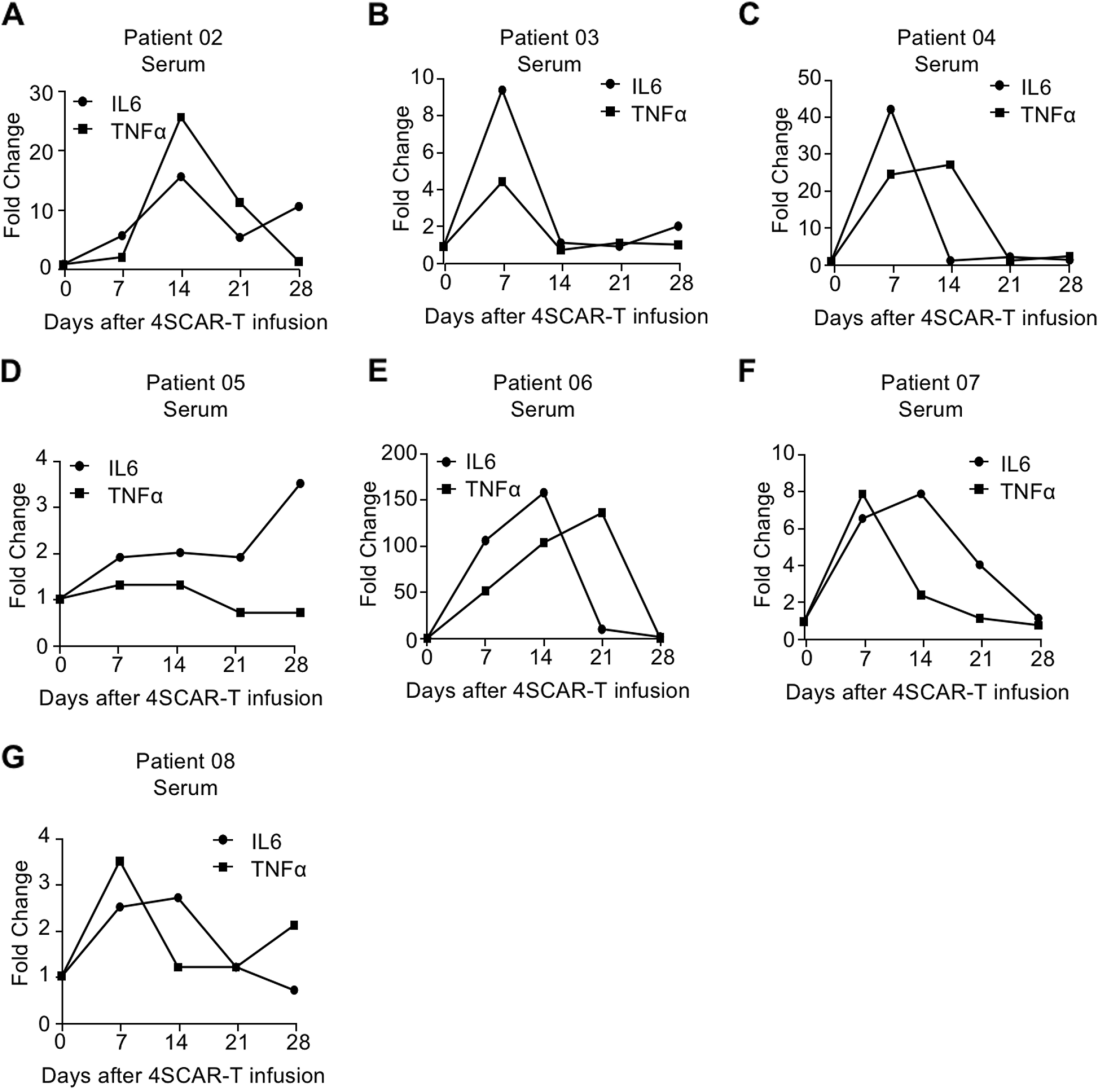
Cytokine modulation in peripheral blood after GD2 specific 4SCAR-T cell infusion. (**A**-**G**) Circulating levels of IL-6 and TNF-α in serum from indicated patients

In addition, we measured circulating IL-6 and TNFα levels in the peripheral blood of the other seven patients infused with 4SCAR-T cells. All patients had elevated levels of IL-6 and TNFα in the peripheral blood after 4SCAR-T cell infusion (Fig. 6A-G). Four of the eight patients had 10-fold or higher elevations in IL-6 and TNFα levels (Figs. 5D and 6A, C, E). Collectively, these findings illustrate that GD2-specific 4SCAR-T cells partially mediate antigen loss and activate immune responses in the tumor microenvironment.

## Discussion

We conducted an in-human pilot study of 4SCAR-T cells targeting GD2 in eight patients with GD2 positive GBM. In this study, the safety of autologous GD2-specific 4SCAR-T cells was assessed in eight patients with progressive GBM. No severe adverse effects were observed in the patients receiving GD2-specific 4SCAR-T cell infusion. CAR-T cells expanded and peaked at 1–3 weeks after infusion. Of the eight evaluable patients, five patients had a clinical benefit as defined by a partial response and stable disease. Three patients with clinical benefits were alive after more than 12 months of follow-up. The median overall survival of this study was 10 months after 4SCAR-T cell infusion (range, 3–24 months).

The trafficking of CAR-T cells to tumors is a major challenge for GBM treatment, and the route of infusion appears to be important. Reports show that different infusion routes including intravenous, intracavitary, and intraventricular injections have been attempted for GBM CAR-T therapies, although intravenous injection is commonly used. Although intracavitary and intraventricular injections require implantation of the Rickham device, these routes enable the trafficking of CAR-T cells into tumor sites of the central nervous system [18, 32]. A previous study performed intracavitary and intraventricular injection of CAR-T cells (IL-13Ra2-specific) in patients with progressive GBM [18]. Both intracavitary and intraventricular infusions of IL-13Ra2-specific CAR-T cells were well tolerated [18]. In addition, intravenous and intraventricular infusions of GD2-specific CAR-T cells for patients with GBM were also well tolerated [24]. In our case, in addition to intravenous infusion of GD2-specific 4SCAR-T cells, three patients were infused with 4SCAR-T cells into the resected tumor cavity at the same time. Intravenous infusion alone or intravenous infusion combined with intracavitary infusion was well tolerated without severe adverse effects. However, a definitive conclusion on which infusion route is better cannot be made from our study due to the small sample size. Because of the limited number of patients, the comparative studies between these two infusion routes on CAR-T cell expansion and anti-tumor functions were not feasible. Therefore, further studies with larger sample size are required.

The major observation from this study was the effect of 4SCAR-T cells on the tumor immune microenvironment. We observed that there were a large number of T cells and macrophages infiltrating the tumor resected after GD2-specific 4SCAR-T cell infusion. In situ phenotypic analysis of post-infusion T cells showed that the infiltrated T cells were CD8 + T cells. In addition, fewer CD163 + M2 macrophages were detected in post-infusion tumor specimens. These findings suggest that infused 4SCAR-T cells stimulate the immune response in the tumor microenvironment.

Infused GD2-specific 4SCAR-T cells could potentially recognize GD2 antigen expressed in GBM and exert tumor-killing functions. In one patient whose tumor was resected 6 weeks after infusion, we found that GD2 antigen expression dramatically decreased in GBM specimens post-infusion. The up-regulated expression of GD2 antigen in cancer cells was reported to be related with NF-κB and its biosynthesis was regulated by GD3 synthase (ST8SIA1), especially in the model of triple-negative breast cancer [20, 33, 34]. GD2 promotes tumor growth and metastasis by cooperating with integrin β1 in melanoma cells [35]. However, little is known on how GD2 expression is down-regulated and how immune responses are activated in the GBM microenvironment after GD2-specific 4SCAR-T cell infusion. Studies from anti-GD-2 therapy in neuroblastoma models showed significant synergy of anti-GD2 and anti-CD47 antibodies treatment, and anti-GD2 blocks the interaction of GD2 with its ligand Siglec-7, an inhibitory immune receptor expressed on human macrophages and NK cells, which then primes neuroblastoma cells for removal by the immune system [36]. Moreover, the effect of anti-GD-2 efficacy in neuroblastoma is associated with an immunosuppressive tumor microenvironment that contains more tumor-associated macrophages and fewer tumor-infiltrating NK cells [37]. In our study, a large number of infiltrated T cells were observed after GD2-specific 4SCAR-T cell infusion. Instead, Lewis Y and CLL1 became the dominant antigens in the GBM of the same patient. These findings provide strong evidence for antigen loss and tumor editing. Therefore, CAR-T cells targeting multiple antigens as well as in combination with immunocytokines, like IL-15, will be an important future direction [23, 38, 39].

Despite these advances, there are several limitations. MRI is a standard approach for assessing tumor progression and response in GBM. However, we observed that MRI could not effectively distinguish pseudo-progression and true disease progression in this study. In our case, we observed immune cell infiltration and inflammation in the tumor after 4SCAR-T administration, which often resulted in pseudoprogression and could affect the assessment of tumor progression. A similar observation was reported in a previous clinical study, which showed infused EGFRvIII-specific CAR-T cells in patients with progressive GBM [17]. Therefore, advanced techniques are required to assess the anti-tumor effect and tumor progression in patients undergoing CAR-T therapy in the future.

The sample size of our study is limited, thus definitive conclusions on the clinical benefits cannot be drawn yet. A previous study demonstrated that children (< 18 years) with high-grade glioma have a better prognosis than adults [40]. In this regard, enrollment of children in our study might have affected the results and conclusions. In addition, measurement of 4SCAR-T cells in CSF would have been informative, but we were unable to determine it owing to the low frequency of CAR-T cells in CSF.

## Conclusion

In conclusion, our study illustrates that infusion of autologous GD2-specific 4SCAR-T cells into patients with GBM via two different routes is safe and well-tolerated. In addition, GD2-specific 4SCAR-T cells partially mediate antigen loss and activate immune responses in the tumor microenvironment. Although extended lifespan and specific antigen loss of GD2 were observed in some patients, the clinical benefit could not be determined from this study due to the small sample size. Our initial clinical trial highlights the safety of GD2-specific 4SCAR-T cells in targeting GBM, and further phase 2 studies evaluating GD2-specific 4SCAR-T cells are warranted.

## Data Availability

All data generated during this study are included in this published article and are available from the corresponding author on reasonable request.

## Abbreviations

4SCAR: Fourth-generation safety-designed chimeric antigen receptor
GBM: Glioblastoma
CAR: Chimeric antigen receptor
IL: Iterleukin
EGFRvIII: Epidermal growth factor receptor variant III
HER2: Human epidermal growth factor receptor 2
DIPG: Diffuse intrinsic pontine glioma
CSC: Cancer stem cell
H3K27M: K27M mutation in genes encoding histone H3
DMG: Diffuse midline glioma
IHC: Immunohistochemistry
PR: Partial response
PD: Progressive disease
SD: Stable disease
PBMC: Peripheral blood mononuclear cells
MRI: Magnetic resonance imaging
